# Exploring of gut microbiota features in dyslipidemia and chronic coronary syndrome patients undergoing coronary angiography

**DOI:** 10.3389/fmicb.2024.1384146

**Published:** 2024-04-05

**Authors:** Wongsakorn Luangphiphat, Pinidphon Prombutara, Viroj Muangsillapasart, Damrong Sukitpunyaroj, Eric Eeckhout, Malai Taweechotipatr

**Affiliations:** ^1^Innovative Anatomy Program, Faculty of Medicine, Srinakharinwirot University, Bangkok, Thailand; ^2^Division of Cardiology, Department of Medicine, Chulabhorn Hospital, Chulabhorn Royal Academy, Bangkok, Thailand; ^3^Princess Srisavangavadhana College of Medicine, Chulabhorn Royal Academy, Bangkok, Thailand; ^4^Omics Sciences and Bioinformatics Center, Faculty of Science, Chulalongkorn University, Bangkok, Thailand; ^5^Mod Gut Co., Ltd., Bangkok, Thailand; ^6^Service of Cardiology, Lausanne University Hospital and University of Lausanne, Lausanne, Switzerland; ^7^Center of Excellence in Probiotics, Srinakharinwirot University, Bangkok, Thailand; ^8^Department of Microbiology, Faculty of Medicine, Srinakharinwirot University, Bangkok, Thailand; ^9^Clinical Research Center, Faculty of Medicine, Srinakharinwirot University, Ongkharak, Thailand

**Keywords:** coronary artery disease, dyslipidemia, gut microbiome, gut microbiota, chronic coronary syndrome, cardiovascular disease

## Abstract

Chronic coronary syndrome (CCS) has a high mortality rate, and dyslipidemia is a major risk factor. Atherosclerosis, a cause of CCS, is influenced by gut microbiota dysbiosis and its metabolites. The objective of this study was to study the diversity and composition of gut microbiota and related clinical parameters among CCS patients undergoing coronary angiography and dyslipidemia patients in comparison to healthy volunteers in Thailand. CCS patients had more risk factors and higher inflammatory markers, high-sensitivity C-reactive protein (hs-CRP) than others. The alpha diversity was lower in dyslipidemia and CCS patients than in the healthy group. A significant difference in the composition of gut microbiota was observed among the three groups. The relative abundance of Proteobacteria, Fusobacteria, Enterobacteriaceae, *Prevotella,* and *Streptococcus* was significantly increased while *Roseburia*, *Ruminococcus,* and *Faecalibacterium* were lower in CCS patients. In CCS patients, Lachnospiraceae, Peptostreptococcaceae, and *Pediococcus* were positively correlated with hs-CRP. In dyslipidemia patients, *Megasphaera* was strongly positively correlated with triglyceride (TG) level and negatively correlated with high-density lipoprotein cholesterol (HDL-C). The modification of gut microbiota was associated with changes in clinical parameters involved in the development of coronary artery disease (CAD) in CCS patients.

## Introduction

Cardiovascular disease (CVD) is the world’s leading cause of mortality and one of Thailand’s significant health issues. This disease has several risk factors, including dyslipidemia (Hedayatnia et al., 2020), diabetes mellitus (Abdul-Ghani et al., 2017), hypertension (Dustan, 2000), obesity, insulin resistance, and metabolic syndrome (Tune et al., 2017). Currently, lifestyle changes like exercising, eating healthily, and consuming less salt to lower blood pressure can help to reduce the risk factors of CVD. These changes can be combined with blood-sugar-lowering drugs, statins, and antihypertensive drugs. However, morbidity and mortality rates, which are still very high all over the world, including Thailand, are impacted by CVD.

Even though there are other coronary artery disease (CAD) risk factors, dyslipidemia is a major one (Yusuf et al., 2004). Acute and chronic coronary syndromes are two subtypes of CAD that nare distinguished by the timing of the onset of the signs and symptoms. A phrase used to describe CAD as a chronic, progressive condition that can be stabilized is chronic coronary syndrome (CCS) (Knuuti et al., 2020).

More than 1,100 different bacterial species have been linked to numerous symptoms and diseases, including cancer, diabetes mellitus, obesity, and CVD (Afzaal et al., 2022), according to earlier research. Since metabolic syndrome is a cardiovascular condition, eating the proper amount of healthful food can both prevent and treat it. The majority of gastrointestinal tract microorganisms cannot be cultured. The genes of the microorganism, also known as the microbiome, must therefore be examined to determine the type and quantity of these microorganisms.

Gut microbiota is a microorganism that lives in the human digestive tract. The quantity and type of microorganisms in the gastrointestinal tract are determined and influenced by several external factors such as geographic origin, age, genetics, diet, and use of prebiotics and antibiotics (Thursby and Juge, 2017; Fan and Pedersen, 2021). The gastrointestinal tract contains a variety of microorganisms such as Firmicutes, Bacteroidetes, Actinobacteria, Proteobacteria, and Fusobacteria, in which their mechanisms of action and habitats are different. Crucial gastrointestinal bacteria that have been presented since birth include *Lactobacillus* and *Bifidobacterium* (Yang et al., 2019). Nowadays people pay attention to the diversity of gut microbiota, which affects the immune system, nutrient metabolism, and the stabilization of the lining of the gastrointestinal tract (gut epithelium). The gut microbiota is also responsible for digesting certain nutrients that cannot be digested in the stomach and also helps to synthesize vitamins and hormones in the body. The body and the gut microbiome have a complicated interaction through metabolites, including short-chain fatty acids (SCFAs), bile acid (BA), and trimethylamine-*N*-oxide (TMAO). Several organs may be affected by these metabolites’ effects. Atherosclerosis, a cause of CVD, is influenced by gut microbiota and metabolites (Pieczynska et al., 2020).

Recent studies have highlighted the role of gut dysbiosis in several diseases, including atherosclerosis and many CVDs. These microorganisms are inherited from the mother at birth, as well as from the environment, food, and medicine (Santacroce et al., 2021). The changing intake of macronutrients leads to metabolic syndrome, if there is an imbalance of microorganisms (dysbiosis) in the gastrointestinal tract. Alterations in gut microbiota will lead to an increase in various diseases and consuming a low-fiber, high-fat, and high-sugar diet affects the digestive system including the gut ecosystem. The increase of the abundance of lipopolysaccharides (LPS)-producing gram-negative bacteria, such as *Escherichia, Shigella, Veillonella, Haemophilus,* and *Klebsiella*, increased with the severity of CAD in contrast to the bacteria that produce butyric acids, such as Ruminococcaceae and Lachnospiraceae, decreased the onset of CAD. Moreover, several types of gut microbiota, including those from Bacteroidetes phylum, *Roseburia intestinalis, Faecalibacterium prausnitzii*, and *Eubacterium rectale*, are also protective factors against CAD (Jonsson and Bäckhed, 2017; Liu H. et al., 2019; Liu et al., 2020).

Sequencing of the 16S rRNA gene is a clustering-independent widely used technique in microbiome studies to analyze the composition and diversity of bacterial communities. This technique provides insights into the diversity, distribution, and relative abundance of bacteria, which are essential for understanding the role of microbiota in various environments, including the human body, soil, water, and other ecological systems (Lane et al., 1985; Wensel et al., 2022).

To the best of our knowledge, no study has been conducted in Thailand to determine if the gut microbiome is associated with the parameters of dyslipidemia and CCS patients undergoing coronary angiography. The purpose of this exploratory study is to identify potential relationships among relative gut microbiota composition and related parameters in patients with dyslipidemia and CCS patients undergoing coronary angiography in Thailand.

## Materials and methods

### Subject enrollment

**Figure 1:**
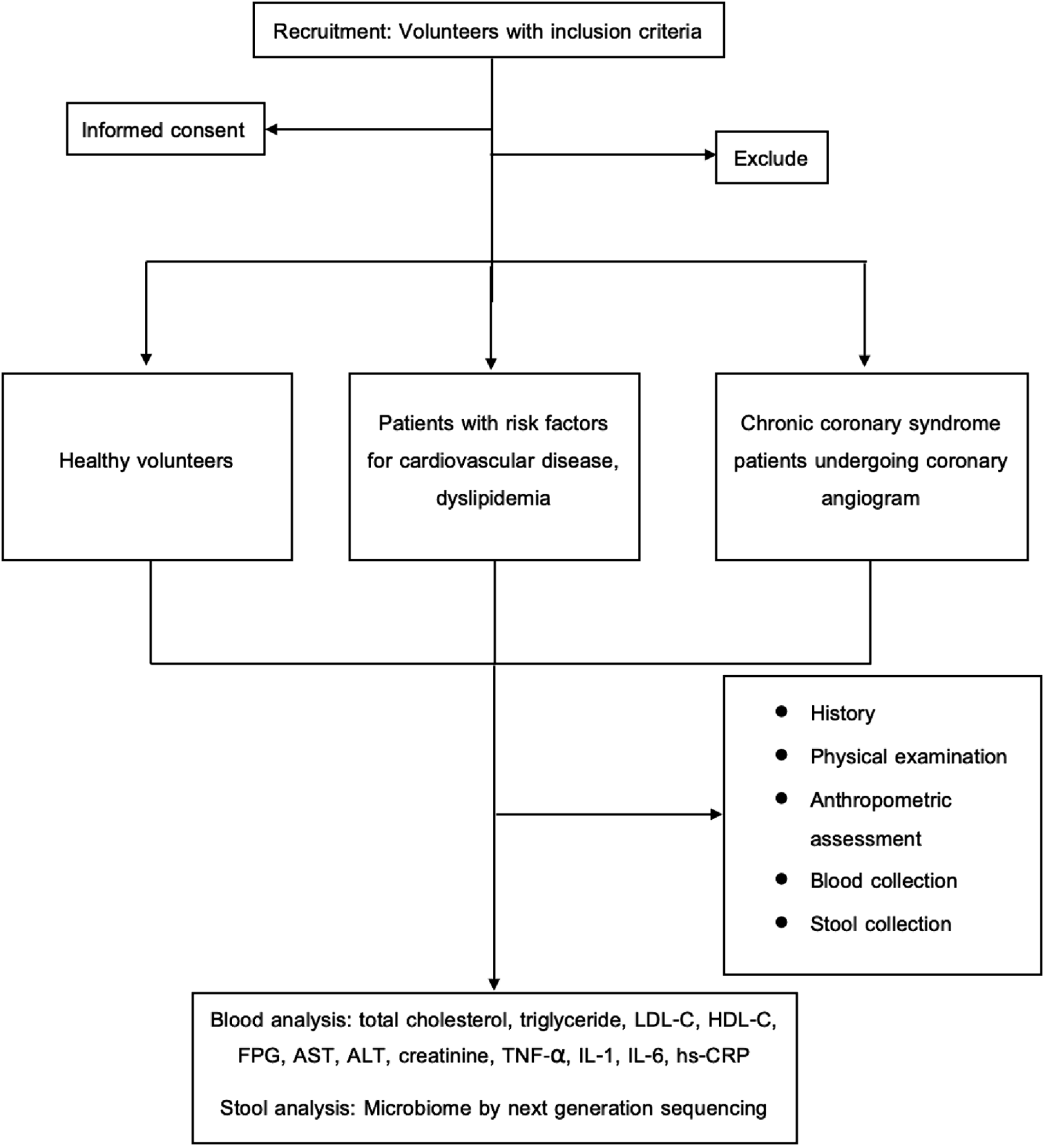
Schematic diagram showing the research workflow. ALT, alanine transaminase; AST, aspartate transaminase; FPG, fasting plasma glucose; HDL-C, high-density lipoprotein cholesterol; hs-CRP, high-sensitivity C-reactive protein; IL-1, interleukin-1; IL-6, interleukin-6; LDL-C, low-density lipoprotein cholesterol; TNF-α, tumor necrosis factor-alpha.

Ninety-one patients between the ages of 35 and 70 who were admitted to Chulabhorn Hospital’s cardiovascular center between February and June of 2023 were enlisted. Using statistical matching techniques with age and sex, participants were separated into three groups: CCS patients undergoing coronary angiography, patients with cardiovascular risk factors, dyslipidemia, and healthy volunteers. This study protocol was approved by the ethics committee of Chulabhorn Hospital. All individuals provided written informed consent before participating in the study. The Schematic diagram showing the research workflow is in Figure 1.

### Inclusion criteria

In patients with CCS undergoing coronary angiography group, they must meet the following criteria: (1) patients with stable anginal symptoms (2) patients <1 year after an acute coronary syndrome (ACS) with stabilized symptoms after revascularization (3) CCS patients >1 year after initial diagnosis or revascularization (4) asymptomatic patients in whom CAD is found after screening (Knuuti et al., 2020). Coronary angiography was used to confirm the diagnosis of CCS in those with ≥70% stenosis in coronary arteries larger than 2.5 mm in one view; ≥ 50% stenosis in coronary arteries in two views; and ≥ 50% stenosis in the left main coronary artery (Jiangping et al., 2013); and they must assent by signing the consent forms.

In the group of dyslipidemia patients, they must only have one risk factor for CVD, dyslipidemia (Total cholesterol (TC) ≥ 200 mg/dL or triglyceride (TG) ≥ 150 mg/dL or low-density lipoprotein cholesterol (LDL-C) ≥ 160 mg/dL or patients on treatment) (Lin et al., 2018), without type 2 diabetes mellitus (T2DM) (fasting blood glucose (FBS) ≥ 126 mg/dL for 2 times separately or patients on treatment) (American Diabetes Association, 2014), hypertension (systolic blood pressure ≥ 140 mmHg and/or diastolic blood pressure ≥ 90 mmHg at least 2 different days or patients on treatment) (Williams et al., 2019), obesity (body mass index ≥30 kg/m2) (Samadoulougou et al., 2020), and metabolic syndrome [at least 3 of the following risk factors: waist circumference ≥ 90 cm for men and ≥ 80 cm for women, blood pressure ≥ 130/85 mmHg or on antihypertensive drugs, TG ≥ 150 mg/dL or on medication, high-density lipoprotein cholesterol (HDL-C) ≤ 40 mg/dL for men and ≤ 50 mg/dL for women and FBS ≥100 mg/dL or on medication] (Huang, 2009). Additionally, none of the aforementioned diseases must exist among the group of healthy volunteers.

### Exclusion criteria

Patients who met the following criteria were excluded: (1) kidney disease (estimated glomerular filtration rate (eGFR) < 60 mL/min/1.73 m2 for more than 3 months), liver disease (Aspartate aminotransferase (AST), Alanine aminotransferase (ALT) > 5 times normal or jaundice), cancer, intestinal diseases such as inflammatory bowel disease, thyroid dysfunction, immunodeficiency such as human immunodeficiency virus (HIV); (2) use antibiotics, immunosuppressants, probiotics supplement, synbiotics, herbal supplements, antacids or laxatives within 4 weeks before participating in the study; (3) history of gastrointestinal disease and other infections within 4 weeks; (4) smoking; (5) alcoholism; (6) pregnant or lactating; (7) Covid-19 infection within 4 weeks.

### Sample size calculation

The sample size calculation is based on “Hypothesis testing and power calculations for taxonomic-based human microbiome data,” in this research, the value of Alpha (α) = 5%, power = 80%, and the function of the number of sequence reads is equal to 50,000 with three groups. A total of 25 volunteers were obtained and it was determined that the dropout rate of 20%, resulting in a total of 30 volunteers per group, or 90 volunteers in total (La Rosa et al., 2012).

### Sample collection and high-throughput sequencing

Each patient’s blood was collected to evaluate FBS, hemoglobin A1C (HbA1C), TC, TG, HDL-C, LDL-C, AST, ALT, creatinine, and high-sensitivity C-reactive protein (hs-CRP). The fresh stool was collected in DNA/RNA shield fecal collection tubes (Zymo Research, CA, United States) 1 day before an appointment and immediately frozen at-20°C for 48 h before further analysis. Using the QIAamp Stool Mini kit (Qiagen, United States), DNA was extracted from stool samples. Nanodrop and electrophoresis were used to evaluate the quantity and quality of DNA. Using 515\u00B0F and 806R primers and 2X KAPA hot-start ready mix, the V4 hypervariable region of the 16S rRNA gene was amplified by PCR. The PCR conditions included an initial denaturation at 94°C for 3 min, followed by 25 cycles of 98°C for 20 s., 55°C for 30 s., 72°C for 30 s., and a final extension step at 72°C for 5 min. The 16S amplicons were purified using AMPure XP beads and indexed using Nextera XT index kit, followed by 8 cycles of the aforementioned PCR condition. Finally, the PCR products were cleaned and pooled for cluster generation and 250-bp paired-end read sequencing on the Illumina^®^ MiSeq^™^.

### Sequencing data analysis

QIIME 22019.10 was used for microbiome bioinformatics (Bolyen et al., 2019). The raw sequence data was demultiplexed using the q2-demux plugin, and reads with expected errors (maxEE) higher than 3.0 were discarded by denoising software, DADA2 (via q2-dada2). A phylogeny was constructed using the SEPP q2-plugin, placing short sequences into sepp-refs-gg-13-8.qza reference phylogenetic tree. Alpha-diversity metric, beta-diversity metric, and Principle Coordinate Analysis (PCoA) were estimated using q2-diversity after samples were rarefied (subsampled without replacement) to a minimum read. Taxonomy was assigned to ASVs using the classify-sklearn naïve Bayes taxonomy classifier against the Greengenes 13_8 99% operational taxonomic units (OTUs) reference sequences. Statistical tests of alpha and beta diversity were performed using Kruskal-Wallis and permutational multivariate analysis of variance (PERMANOVA) (number of permutations = 999), respectively. Moreover, the significantly differential abundance analysis of microbiota was conducted using LEfSe (Segata et al., 2011) via the algorithm module on the Galaxy platform at http://huttenhower.sph.harvard.edu/galaxy. First, nonparametric factorial Kruskal-Wallissum-rank tests were applied to choose features differentially distributed among classes (*p* < 0.05). The linear discriminant analysis (LDA) model was used to estimate their effect sizes and supported by 30-fold bootstrapping (cutoff = logarithmic LDA score of ≥2.0). In addition, significant *p*-values associated with the microbial significantly differential features by LEfSe were corrected for multiple hypothesis testing using the Benjamini and Hochberg false discovery rate correction.

### Statistical analysis and visualization

Stata/SE 16.1 software (StataCorp LP, College Station, TX, United States) was used to analyze the statistical data. Statistics were considered significant when *p*-value <0.05. All study variables were subjected to descriptive statistics analysis, which was provided as frequency (%) for categorical data and mean ± standard deviation (SD) or median for nonnormal quantitative data. One-way analysis of variance (ANOVA) statistic and *post hoc* analysis using the Scheffe test with *p*-value <0.05 were utilized if the distribution of the quantitative data, such as age and laboratory results, was normal. The Kruskal-Wallis test and *post hoc* Mann–Whitney U test with *p*-value <0.017 were used if the data distribution was not normal.

The following Spearman’s correlation coefficient was analyzed; (1) anthropometric measurements, including BMI and waist circumference (2) physical examination; blood pressure and (3) blood tests; FBS, HbA1C, TC, TG, LDL-C, HDL-C, AST, ALT, and hs-CRP. Correlation heat map visualization was performed using the ggplot2 R package. A *p*-value <0.05 was considered statistically significant and was labeled in the figure. In addition, the phylogenetic heat tree was visualized using the metacoder R package.

## Results

### Clinical characteristics

Ninety-one patients were included and divided into three groups: CCS patients undergoing coronary angiography, dyslipidemia patients, and healthy volunteers, with 30, 32, and 29 patients in each group, respectively. To be mentioned, 96 patients were recruited but the microbiota data of one dyslipidemia patient and four CCS patients were missed because stool samples could not be collected. So, we examined 91 samples of the gut microbiota. The patients were 43.96% female, with a mean age of 57.57 ± 8.35 years, and 26.37% had hypertension. There was no statistically significant difference in age or gender among the groups. Baseline characteristics are shown in Table 1.

**Table 1:** Clinical characteristics of the patients (*N* = 91) in this study.

Parameters	Total (*n* = 91)	CCS (*n* = 30)	DLP (*n* = 32)	Healthy (*n* = 29)	*p*-value
Age (years)	57.57 ± 8.35	59.60 ± 9.04	57.44 ± 8.88	55.62 ± 6.60	0.066^b^
Male (%)	51 (56.04)	20 (66.67)	16 (50.00)	15 (51.72)	0.356^c^
BMI (kg/m^2^)	24.10 ± 4.07	24.63 ± 3.47	25.19 ± 5.45	22.37 ± 1.78	0.003^b^
Waist circumference (cm)	82.32 ± 9.76	88.20 ± 8.92	81.25 ± 10.59	77.43 ± 6.04	<0.001^b^
History of CAD (%)	17 (18.68)	17 (56.67)	0 (0.00)	0 (0.00)	<0.001^c^
Medication					
Antiplatelets	30 (32.97)	30 (100.00)	0 (0.00)	0 (0.00)	<0.001^c^
Antihypertensive drugs	24 (26.37)	24 (80.00)	0 (0.00)	0 (0.00)	<0.001^c^
Oral antidiabetic drugs	7 (7.69)	7 (23.33)	0 (0.00)	0 (0.00)	<0.001^d^
Statins	58 (63.74)	29 (96.67)	29 (90.63)	0 (0.00)	<0.001^d^
Statin intensity					<0.001^d^
Low intensity	2 (3.45)	0 (0.00)	2 (6.90)	0 (0.00)	
Moderate intensity	18 (31.03)	2 (6.90)	16 (55.17)	0 (0.00)	
High intensity	38 (65.52)	27 (93.10)	11 (37.93)	0 (0.00)	
Duration ≥ 3 months	54 (93.10)	29 (100.00)	25 (86.21)	0 (0.00)	0.112^d^
Obesity (%)	19 (21.11)	13 (43.33)	6 (19.35)	0 (0.00)	<0.001^c^
Abdominal obesity (%)^*^	21 (23.08)	13 (43.33)	5 (15.63)	3 (10.34)	0.005^c^
Hypertriglyceridemia (%)^**^	18 (19.78)	8 (26.67)	10 (31.25)	0 (0.00)	0.005^c^
Low HDL-C (%)^#^	18 (19.78)	13 (43.33)	5 (15.63)	0 (0.00)	<0.001^c^
Impaired fasting glucose (%)^@^	28 (30.77)	21 (70.00)	3 (9.38)	4 (13.79)	<0.001^c^
Metabolic syndrome (%)	10 (10.99)	10 (33.33)	0 (0.00)	0 (0.00)	<0.001^d^
Hypertension (%)	24 (26.37)	24 (80.00)	0 (0.00)	0 (0.00)	<0.001^c^
Diabetes mellitus (%)	9 (9.89)	9 (30.00)	0 (0.00)	0 (0.00)	<0.001^d^
Dyslipidemia (%)	49 (53.85)	17 (56.67)	32 (100.00)	0 (0.00)	<0.001^c^
Heart failure (%)	1 (1.10)	1 (3.33)	0 (0.00)	0 (0.00)	0.648^d^
Stroke (%)	0 (0.00)	0 (0.00)	0 (0.00)	0 (0.00)	1.000^d^
PAD (%)	1 (1.10)	1 (3.33)	0 (0.00)	0 (0.00)	0.648^d^
SBP (mmHg)	121.65 ± 11.88	124.23 ± 14.34	122.41 ± 10.12	118.14 ± 10.35	0.174^b^
DBP (mmHg)	73.99 ± 9.64	72.27 ± 7.76	73.59 ± 10.57	76.21 ± 10.20	0.283^a^
Laboratory data					
FBS (mg/dl)	99.74 ± 18.81	114.31 ± 23.98	95.59 ± 11.00	89.76 ± 8.53	<0.001^b^
HbA1C (mg/dL)	5.69 ± 0.75	6.04 ± 0.97	5.74 ± 0.50	5.28 ± 0.47	<0.001^b^
Total Cholesterol (mg/dL)	175.76 ± 44.19	144.33 ± 41.24	202.93 ± 43.67	178.28 ± 22.28	<0.001^b^
Triglyceride (mg/dL)	119.26 ± 74.86	135.83 ± 92.69	136.44 ± 76.38	83.17 ± 25.98	0.003^b^
LDL-C (mg/dL)	106.72 ± 39.97	77.11 ± 34.44	129.96 ± 40.78	111.70 ± 21.97	<0.001^b^
HDL-C (mg/dL)	56.31 ± 17.89	45.90 ± 15.08	59.84 ± 18.09	63.17 ± 15.88	<0.001^b^
Serum creatinine (mg/dL)	0.86 ± 0.22	0.98 ± 0.20	0.83 ± 0.21	0.77 ± 0.19	<0.001^b^
AST (IU/L)	22.90 ± 11.23	24.50 ± 11.96	25.19 ± 10.60	18.72 ± 10.29	<0.001^b^
ALT (IU/L)	23.22 ± 15.45	26.10 ± 12.79	25.59 ± 10.94	17.62 ± 20.43	<0.001^b^
hs-CRP (mg/dL)	2.36 ± 2.99	3.46 ± 4.26	2.03 ± 2.05	1.58 ± 1.80	0.043^b^

Categorical variables are expressed as frequency and percentage. Continuous variables are expressed as mean ± standard deviation. ^a^ One-way ANOVA, post hoc test analysis using Scheffe, significant when *p*-value < 0.05, ^b^ Kruskal-Wallis test, post hoc test analysis using Mann–Whitney U test, significant when *p*-value < 0.017 (0.05/3), ^c^ Chi square test, significant when *p*-value < 0.05, ^d^ Fisher’s exact test, significant when *p*-value < 0.05, ^+^Obesity, BMI ≥ 25 kg/m^2^, ^*^Abdominal obesity, waist circumference > 90 cm for male, waist circumference > 80 cm for female, ^**^Hypertriglyceridemia = triglyceride ≥ 150 mg/dL, ^#^Low HDL-C = HDL-C < 40 mg/dL for male, HDL-C < 50 mg/dL for female, ^@^Impaired fasting glucose = FBS ≥ 100 mg/dL, ALT, alanine aminotransferase; AST, aspartate aminotransferase; BMI, body mass index; CAD, coronary artery disease; CCS, chronic coronary syndrome patients; DBP, diastolic blood pressure; DLP, dyslipidemia patients; FBS, fasting blood sugar; HbA1C, hemoglobin A1C; healthy, healthy volunteers; hs-CRP, high-sensitivity C-reactive protein; HDL-C, high-density lipoprotein cholesterol; IU/L, international units per liter; LDL-C, low-density lipoprotein cholesterol; mmHg, millimeters of mercury; mg/dL, milligrams per deciliter, ng/L, nanogram per liter; PAD, peripheral artery disease; SBP, systolic blood pressure.

The average age of the CCS patients was 59.60 ± 9.04 years and 56.67% of them had a history of CAD. In comparison to other groups, more patients had obesity, low HDL-C level, metabolic syndrome, hypertension, and diabetes mellitus at 43.33, 43.33, 33.33, 80, and 30% with statistically significant, respectively. Additionally, the levels of FBS, HbA1C, and hs-CRP were higher in this group than in others with statistical significance.

In dyslipidemia patients, the mean age was 57.44 ± 8.88 years. Diabetes mellitus and hypertension patients were omitted. There were 9.38% of the patients with impaired fasting glucose in this group however the average of FBS and HbA1C was in the normal range. TC and LDL-C were higher than others with statistical significance.

Unexpectedly, TC and LDL-C were found to be lowest in CCS patients compared to patients with dyslipidemia and healthy volunteers at 144.33 ± 41.24 and 77.11 ± 34.44 vs. 202.93 ± 43.67 and 129.96 ± 40.78 vs. 178.28 ± 22.28 and 111.70 ± 21.97, respectively. According to this study, 96.67% of CCS patients utilized statins, with a high-intensity statin rate of 93.10%. All patients took statins for a minimum of three months. Patients with dyslipidemia used statins at a rate of 90.63% with only high-intensity statins at 37.93%.

### Diversity of the gut microbiota

**Figure 2:**
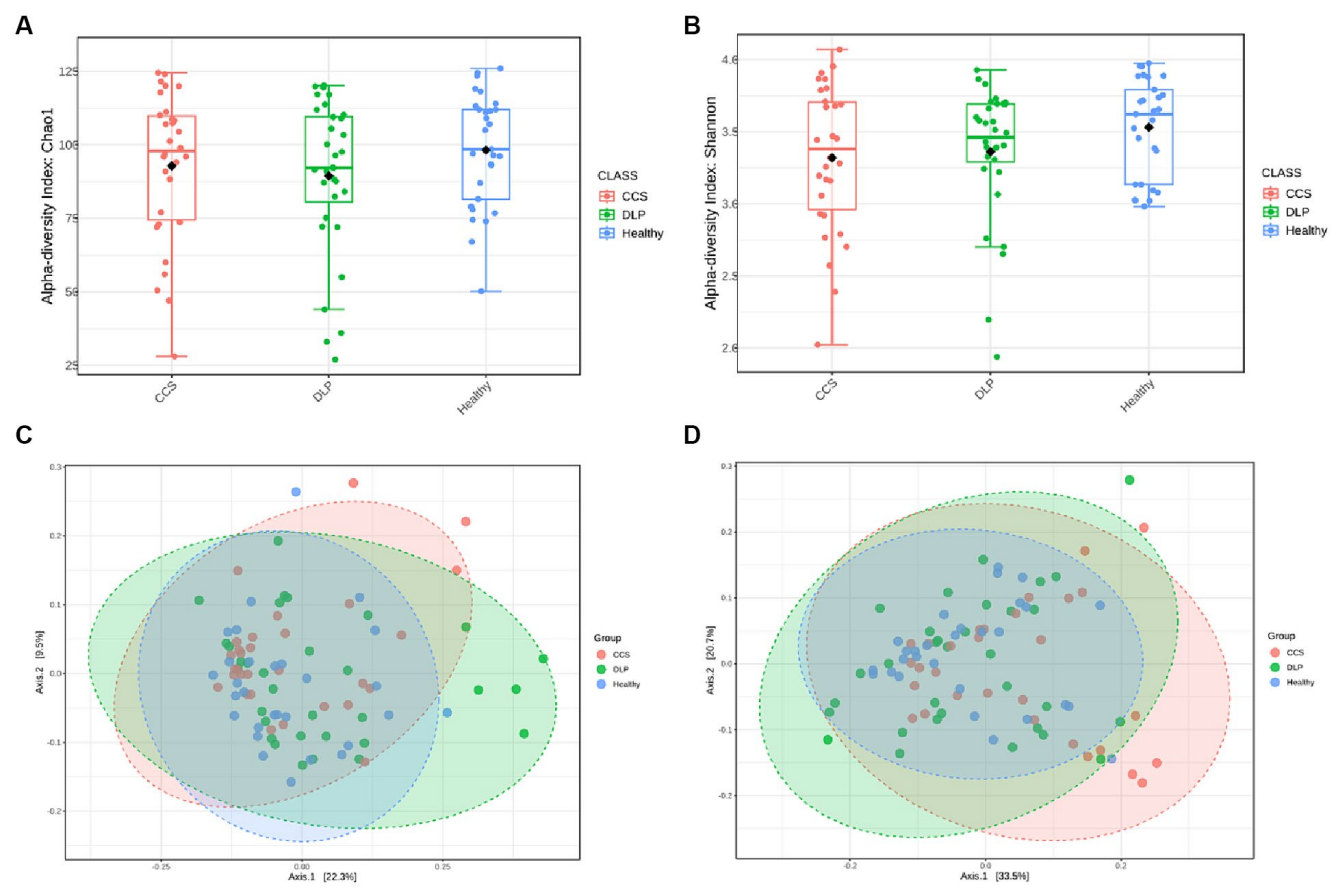
Analysis of alpha- and beta-diversity of microbial composition in the three patient groups. Diversity within bacterial communities was measured by the Chao1 index **(A)**, the Shannon diversity index **(B)** the principal coordinate analysis (PCoA) of beta diversity based on the unweighted UniFrac distance **(C)** and the weighted UniFrac distance **(D)**. Kruskal-Wallis H test was used in the statistical test of the alpha diversity (no statistically significant difference found). Permanova (Permutational multivariate analysis of variance) test was used in the statistical test of the beta diversity. Statistically significant differences found at *p* < 0.001. CCS, chronic coronary syndrome patients; DLP, dyslipidemia patients; Healthy, healthy volunteers.

A bacterial diversity analysis was performed to characterize the microbial community. Chao1 index was estimated to reflect the number of OTUs in a sample, the values of which are positively correlated with the species richness of the sample. Moreover, Shannon indicates it reflects the averaging or uniformity of the abundance of different species in a sample, which is positively correlated with the species diversity. Alpha diversity analyses, including the values of Chao1 and Shannon indexes, are comprehensive indicators of species richness and uniformity in community ecology (Figures 2A,B). From both Chao1 and Shannon indexes, there was a trend that showed lower diversity in dyslipidemia patients and CCS patients than in healthy volunteers.

Beta diversity refers to species differences between different environmental communities, and it also can be used to evaluate the overall heterogeneity of the species or the environmental community. Weighted and Unweighted UniFac distances were used in this study to determine the similarities and differences in the composition structure between the microbial communities. Principal coordinates analysis illustrated a statistically significant difference (PERMANOVA, *p* < 0.01) in the clustering of fecal samples between the gut microbiome of the dyslipidemia patients, CCS patients, and healthy volunteers (Figures 2C,D). The different colors in the PCoA analysis represent different samples, and the different shapes represent different groups. The closer the sample distance is, the more similar the microbial composition between the samples is, and the smaller the difference is.

### Assessment of bacterial taxonomic composition of CCS and dyslipidemia patients and healthy volunteers

**Figure 3:**
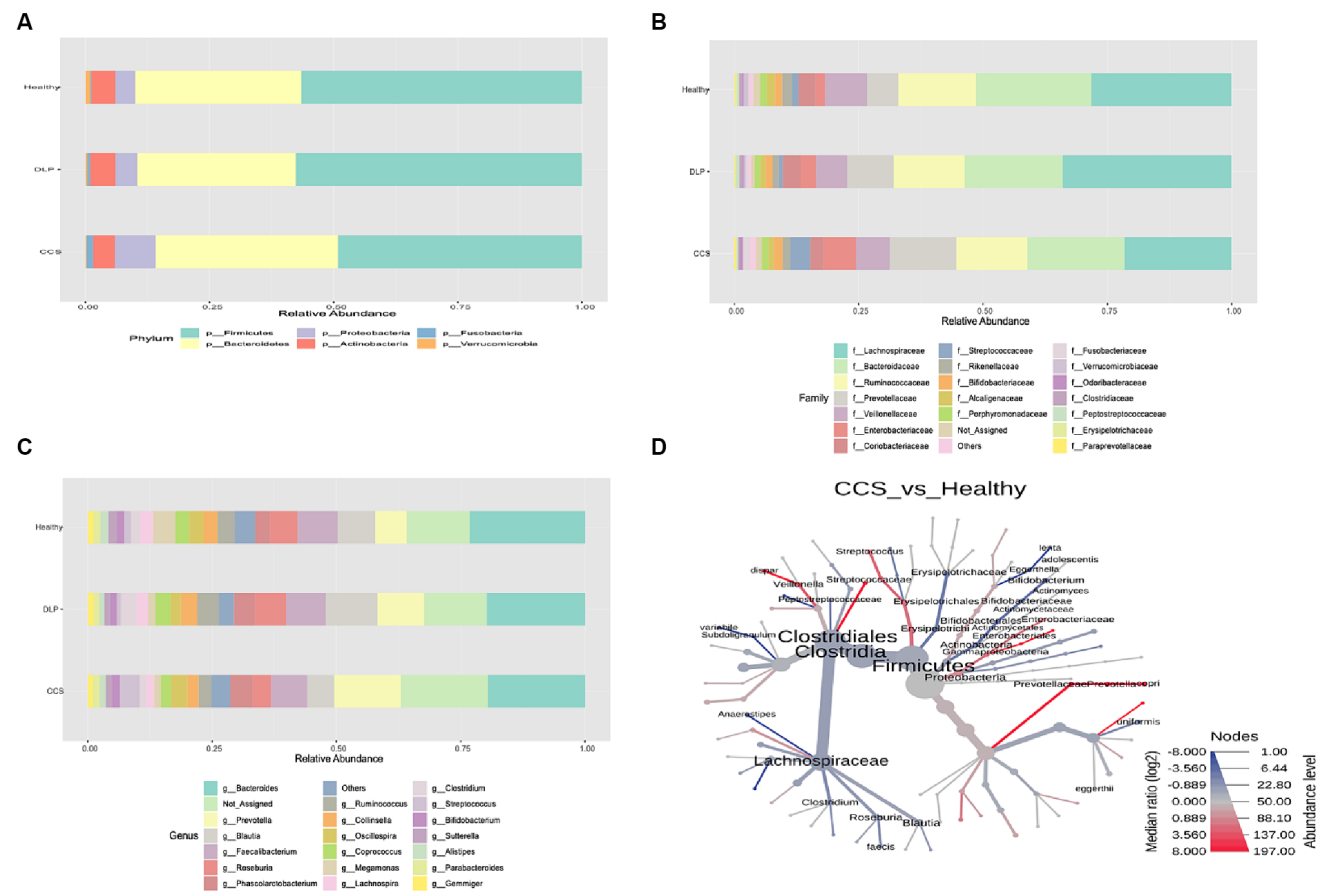
The relative abundance of bacterial taxonomic profile in the feces at the phylum **(A)** family **(B)** and genus **(C)** level. The phylogenetic heat tree **(D)** in comparison of bacterial microbiota between CCS and healthy groups shows the bacteria composition at the species level. The color of nodes and edges represents the mean change in operational taxonomic units (OTUs) richness at each taxonomic group, with red indicating greater richness in CCS and blue in healthy, while node size represents OTUs richness of each taxonomic group across the dataset. CCS, chronic coronary syndrome patients; DLP, dyslipidemia patients; Healthy, healthy volunteers.

At the phylum level, among all three groups, the most common bacterial phyla were Firmicutes, Bacteroidetes, Proteobacteria, and Actinobacteria. The relative abundance of Proteobacteria was increased in CCS patients (the percentage of Proteobacteria in CCS patients, dyslipidemia patients, and healthy volunteers: 8.22, 4.79, and 4.16, respectively). Moreover, the relative abundance of Fusobacteria was also increased in CCS patients (the percentage of Fusobacteria in CCS patients, dyslipidemia patients, and healthy volunteers: 1.48, 1.07, and 0.89, respectively). On the other hand, in CCS patients, Actinobacteria and Verrucomicrobia had the lowest relative abundance proportion among the three groups. In terms of other phyla, there was no difference. The proportion of Firmicutes and Bacteroidetes was higher in the group of dyslipidemia patients than in the other groups (Figure 3A).

At the family level, when the gut microbiota of the three groups was analyzed at this level, Lachnospiraceae and the Bacteroidaceae families were shown to be the two most common families. Compared to other groups, Prevotellaceae, Enterobacteriaceae and Streptococcaceae families were more prevalent proportionately in CCS patients (Figure 3B).

At the genus level, the highest abundance in each group belonged to the genus *Bacteroides* (the percentage of *Bacteroides* in CCS patients, dyslipidemia patients, and healthy volunteers: 19.02, 19.15, and 21.50, respectively). In the group of CCS patients, *Prevotella* and *Streptococcus* genera were more prevalent in abundance than other groups, with statistical significance following from *Bacteroides* genus (Figure 3C). Moreover, the relative abundance of *Roseburia* (the percentage of *Roseburia* in CCS patients, dyslipidemia patients and healthy volunteers: 3.17, 5.67, and 4.71, respectively), *Ruminococcus* (the percentage of *Ruminococcus* in CCS patients, dyslipidemia patients and healthy volunteers: 2.40, 4.54, and 3.79, respectively) and *Faecalibacterium* genera (the percentage of *Faecalibacterium* in CCS patients, dyslipidemia patients and healthy volunteers: 6.20, 6.98 and 7.08, respectively) were the lowest in CSS patients’ group than in the others.

At the species level, from the pairwise phylogenetic heat tree, the abundance of *Streptococcus, Veillonella dispar,* and *Prevotella copri* was higher in CCS patients than in healthy volunteers with statistical significance. On the other side, fewer *Roseburia faecis*, *Subdoligranulum* var*iabile, Bifidobacterium,* and *Anaerostipes* were detected in this group (Figure 3D).

### The characteristic of bacterial microbiota of CCS patients undergoing coronary angiography

**Figure 4:**
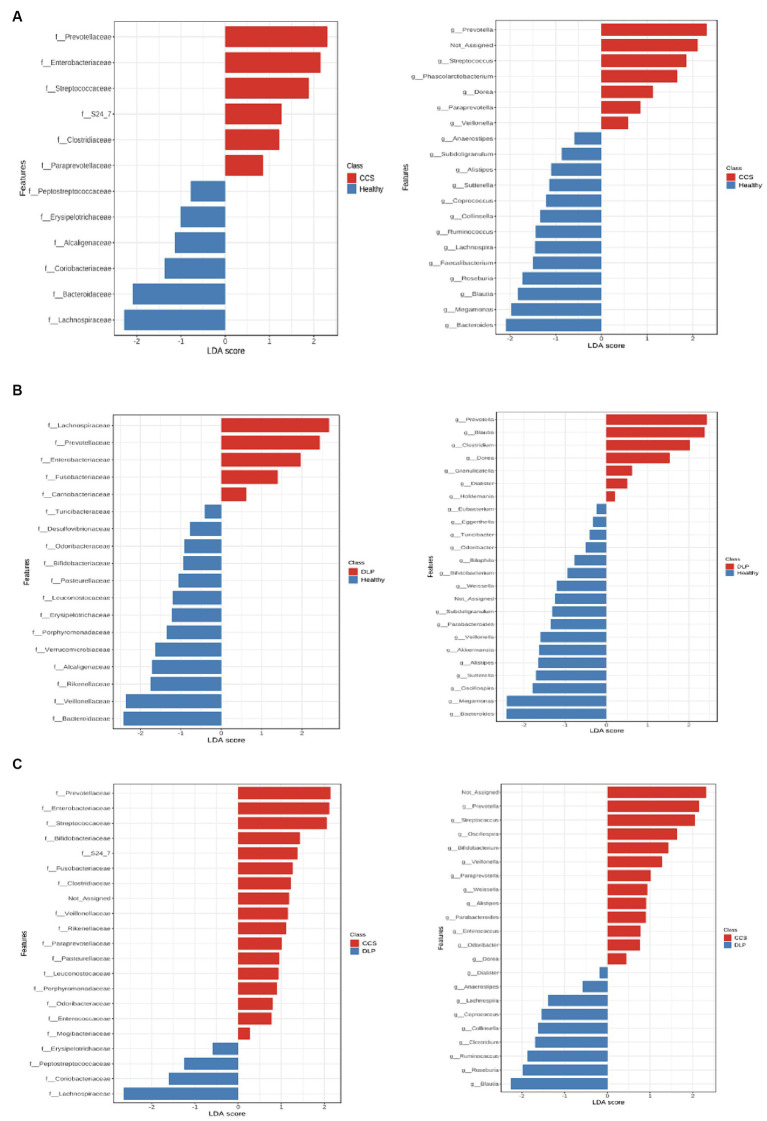
The linear discriminant analysis effect size (LEfSe) in comparison of bacterial microbiota between CCS patients and healthy volunteers **(A)**, dyslipidemia patients and healthy volunteers **(B)**, and CCS patients and dyslipidemia patients **(C)**. CCS, chronic coronary syndrome patients; DLP, dyslipidemia patients; Healthy, healthy volunteers.

In CCS patients, the relative abundance of Prevotellaceae, Enterobacteriaceae, Streptococcaceae, Clostridiaceae, and Paraprevotellaceae families had significantly risen at the family level compared with healthy volunteers, according to the linear discriminant analysis effect size (LEfSe). At the genus level, the LEfSe revealed that *Prevotella, Streptococcus, Phascolarctobacterium, Dorea, Paraprevotella,* and *Veillonella* genera had significantly higher relative abundances in CCS patients compared to healthy volunteers (Figure 4A).

When comparing CCS patients with dyslipidemia patients, The result showed that the relative abundance of Prevotellaceae, Enterobacteriaceae, Streptococcaceae, Fusobacteriaceae, Clostridiaceae, Veillonellaceae, and Porphyromonadaceae families were higher in CCS patients (Figure 4C).

### The characteristic of bacterial microbiota of dyslipidemia patients

In dyslipidemia patients, the relative abundance of Prevotellaceae, Enterobacteriaceae, and Fusobacteriaceae families had significantly risen at the family level compared with healthy volunteers, according to the LEfSe. At the genus level, *Prevotella, Clostridium,* and *Dorea* genera had significantly higher relative abundances in dyslipidemia patients compared to healthy volunteers (Figure 4B).

When we compared dyslipidemia patients with CCS patients, we found that the relative abundance of the Lachnospiraceae family was higher in dyslipidemia patients (Figure 4C).

### Associations of risk factors with gut microbiome composition in CCS patients undergoing coronary angiography

**Figure 5:**
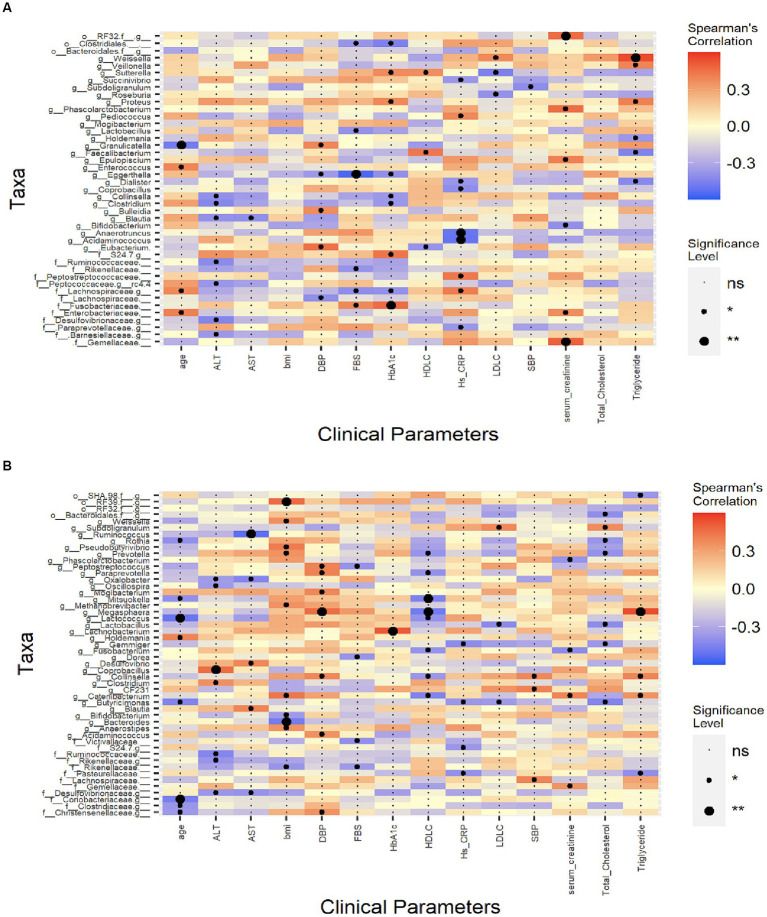
Spearman’s correlation analysis between the clinical indexes and the microbiota. CCS patients undergoing coronary angiography group **(A)**, dyslipidemia patients’ group **(B)**. The colour represents positive (red) or negative (blue) correlations, and *p < 0.05, **p < 0.01. ALT, alanine aminotransferase; AST, aspartate aminotransferase; BMI, body mass index; DBP, diastolic blood pressure; FBS, fasting blood sugar; HbA1C, hemoglobin A1C; hs-CRP, high-sensitivity C-reactive protein; HDL-C, high-density lipoprotein cholesterol; LDL-C, low-density lipoprotein cholesterol; SBP, systolic blood pressure.

We calculated the Spearman correlation coefficient between a range of clinical indicators that may be associated with the risk factors of CCS patients (Table 1) and gut microbiota at the genus level (Figure 5A). We found that Lachnospiraceae, Peptostreptococcaceae families, and *Pediococcus* genus were positively correlated with hs-CRP. *Weissella* genus was positively correlated with LDL-C while the *Sutterella* and *Roseburia* genera were negatively correlated with LDL-C.

### Associations of risk factors with gut microbiome composition in dyslipidemia patients

In dyslipidemia patients, *Pseudobutyrivibrio, Catenibacterium, Weissella, Prevotella,* and *Anaerostipes* genera were positively correlated with body mass index (BMI) while *Bacteroides* and *Bifidobacterium* genera were negatively correlated with BMI. For this group’s lipid profile, genus *Subdoligranulum* was positively correlated with TC level; genus *Megasphaera* was strongly positively correlated and genera *Collinsella* and *Catenibacterium* were also positively correlated with TG level; genus *Megasphaera* was negatively correlated with HDL-C; genus *Subdoligranulum* was positively correlated with LDL-C level and genera *Lactobacillus* and *Butyricimonas* were negatively correlated with LDL-C level (Figure 5B).

## Discussion

This is the first investigation on gut microbiota and associated factors in CCS patients undergoing coronary angiography in Thailand. In this study, the gut microbiome differed among the three groups. There was a reduction in diversity in dyslipidemia patients and CCS patients’ group, which correlated with other studies (Huttenhower et al., 2012; Fu et al., 2015; Sawicka-Smiarowska et al., 2021). This finding is consistent with other studies that patients suffering from various diseases have also reduced bacterial diversity, for instance, hypertension (Yang et al., 2015), Crohn’s disease (Hansen et al., 2012), psoriatic arthritis (Scher et al., 2015), metabolic syndrome (Arora and Bäckhed, 2016), diabetes mellitus, and obesity (Pascale et al., 2019).

In this study, the composition pattern of the bacterial microbiota differed significantly between the group of CCS patients and healthy volunteers. The most prevalent bacterial phyla were Firmicutes, Bacteroidetes, Proteobacteria, and Actinobacteria, which correlated with other studies (Zheng et al., 2020). Alterations in the ratio of the major phyla Firmicutes to Bacteroidetes have been proposed as a potential CAD risk factor, suggesting that changes in the microbiome’s composition may also contribute to the onset and progression of atherosclerosis and CAD (Emoto et al., 2016, 2017; Cui et al., 2017). Furthermore, it was established that a greater Firmicutes/Bacteroidetes ratio (F/B ratio) plays a significant impact on CAD patients (Cui et al., 2017). The F/B ratio is used to diagnose gut dysbiosis and the relationship between this ratio and numerous well-known cardiovascular risk factors, including age, sex, food, and BMI, has been demonstrated (Mariat et al., 2009; Holscher et al., 2015; Emoto et al., 2016; Canfora et al., 2017). However, in this study, dyslipidemia patients had the greatest F/B ratio whereas the CCS patients’ group did not.

Numerous pathogenic genera, including *Escherichia, Salmonella, Vibrio, Yersinia*, and *Legionella,* are members of the phylum Proteobacteria. In our study, the relative abundance proportion of Proteobacteria was the highest in CCS patients. According to Shin et al. study, a higher relative abundance of Proteobacteria is associated with gut dysbiosis and many diseases (obesity, T2DM, and cancers) in human (Shin et al., 2015). Mainly Proteobacteria, particularly *Enterobacteriaceae* and some Firmicutes is the abundance of bacteria producing TMAO precursor (Via et al., 2019).

Fusobacteria proportion was increased in the group of CCS patients, however, Actinobacteria and Verrucomicrobia exhibited the lowest relative abundance proportion in CCS patients in comparison with others. This finding is consistent with the study by Zhang et al. (2019) and Cui et al. (2017). *Fusobacterium nucleatum* may initially cause periodontal disease, which then causes CAD due to atherosclerosis via inflammation and lipid metabolism (Zhou et al., 2022). However, no research on the association between *Fusobacterium* and CAD has been published.

In addition, *Streptococcus, Veillonella,* and *Prevotella* genera were more common in abundance than other groups in the group of CCS patients, with statistical significance following from the *Bacteroides* genus. Previous studies have reported that *Prevotella* and *Streptococcus* genera had a close relationship with metabolic syndrome, atherosclerosis, and CAD (Abranches et al., 2009; Yakob et al., 2011; Madhogaria et al., 2022; Bazaz et al., 2023). It suggested that the changes in the abundance of *Streptococcus, Veillonella, Prevotella* were the characteristics of the bacterial microbiota of the CCS patients. The greatest associations were found for *Streptococcus anginosus* and *Streptococcus oralis*, according to Sayols-Baixeras et al.’s study of the correlation between *Streptococcus* spp. and subclinical coronary atherosclerosis (Sayols-Baixeras et al., 2023). The prevalence of various LPS-producing gram-negative bacteria, including *Escherichia, Shigella, Veillonella, Klebsiella,* and *Haemophilus* increased with the severity of the CAD, according to a study by Liu H. et al. (2019). High blood LPS levels were associated with a threefold increased risk of incident atherosclerosis (Mayr et al., 2006). *Veillonella* can be found in atherosclerosis plaque and is associated with cholesterol levels (Koren et al., 2011). In addition, *Veillonella* has a high relative abundance in CAD patients (Zhang et al., 2019; Bouzid et al., 2022). These findings suggested that the differences in bacterial abundance between the three groups may be linked to the progression of CAD and lipid metabolism in dyslipidemia patients.

The role of *Prevotella* in human health is controversial. *Prevotella* is a beneficial microbe, however, it is associated with chronic inflammation. *Prevotella* is linked to high complex carbohydrate diets from plants, fruits, and vegetables, whereas *Bacteroides* is connected to fat and protein diets (Wu et al., 2011)*. Prevotella* can be found in healthy humans and is considered a commensal bacteria. According to De Filippis et al.’s research, vegetarians had the Mediterranean diet at a high level, which was connected with this bacteria strains and higher levels of SCFAs (De Filippis et al., 2016). According to Emoto et, *Lactobacillus, Streptococcus*, and *Enterococcus* increased in the gut microbiota of CAD patients whereas *Bacteroides* and *Prevotella* decreased (Emoto et al., 2016).

*Prevotella* strains’ genetic diversity may help to explain the variations in how they react to dietary and health conditions in different patients (Ley, 2016). For example, the study of Italian people’s gut microbiome showed that varying dietary choices could be responsible for *P. copri* strains with different functions, which resulted in different ways for human health (Filippis et al., 2019). *P. copri* increases the prevalence in non-Westernized people. They typically consume diets rich in fresh vegetables and fruits (Tett et al., 2019).

However, recent studies have connected increasing *Prevotella* abundance and certain strains to metabolic syndrome, obesity, hypertension, insulin resistance, non-alcoholic fatty liver disease (NAFLD), and low-grade systemic inflammation (Zhu et al., 2013; Pedersen et al., 2016; Madhogaria et al., 2022) due to augmentation mucosal helper T-cell (Th17) immune responses and stimulation epithelial cells to produce interleukin-1 (IL-1), IL-8, IL-6 and IL-23 (Larsen, 2017).

*Prevotella copri* was higher in CCS patients than in healthy volunteers in our study, which is correlated with many studies. Numerous diseases are linked with this microorganism to chronic inflammatory processes; for example, rheumatoid arthritis, periodontitis, HIV infection, metabolic syndrome, inflammatory bowel disease, CAD, and cardiac valve calcification. This bacterium has a role in the development of rheumatoid arthritis (RA) and is an immune-relevant (Pianta et al., 2017). In RA patients, gut dysbiosis may have a role in the early stage of RA as shown by the enrichment of *P. copri* (Alpizar-Rodriguez et al., 2019). Moreover, *P. copri* showed a high degree of genetic and functional diversity depending on the lifestyle of the patients and was associated with worse arthritis in these patients (Nii et al., 2023). In CVD patients, *P. copri* may be a major risk factor, particularly in cardiac valve calcification patients. This microorganism and LDL-C have a positive correlation, which likely supports its pro-inflammatory effects. It is a potential key pathogen implicated in CVDs because of its roles in immunity and inflammation (Liu Z. et al., 2019). Moreover, *P. intermedia, P. nigrescens* were periodontopathic bacteria in atherosclerotic plaques (Gaetti-Jardim et al., 2009).

In this investigation, *Faecalibacterium* genera were lower in the CSS patients’ group than in the others. This finding is correlated with Zhu et al. (2016), *Faecalibacterium, Subdoligranulum, Roseburia*, and *Eubacterium rectale* were decreased. There is a significant anti-inflammatory property of *Faecalibacterium* (Machiels et al., 2014).

The Bacteroides genus had the highest relative abundance when we compared the healthy volunteers’ group to the other groups, and the relative abundance of the Roseburia genus was higher in the healthy volunteers’ group than in the other groups in our study. Due to their ability to produce SCFAs, prior research indicated that Bacteroides and Bifidobacterium have a certain protective effect on metabolism and are primarily protective bacteria for CCS (Hoving et al., 2018; Kazemian et al., 2020; Abdi et al., 2022). In addition, Roseburia has been linked to improved glucose intolerance and weight loss in mice and in patients with atherosclerosis with comparatively high amounts of Collinsella, whereas the normal control group has a substantially larger abundance of Roseburia and Eubacterium (Zhu et al., 2018; Liu H. et al., 2019). A study by Liu H. et al. (2019) revealed that the prevalence of bacteria that produced butyric acid, such as Lachnospiraceae and Ruminococcaceae, decreased with the progression of CAD, this findings are correlated with our study that Lachnospiraceae and Ruminococcacea families were less proportion in CCS patients when compared to others.

SCFAs are metabolites of the fermentation of complex carbohydrates. Members of phylum Bacteroidetes can produce butyrate and acetate, whereas phylum Firmicutes can produce butyrate. SCFAs have a favorable correlation with *Roseburia, Bacteroides* spp., and *Eubacterium rectale*. They have been proposed as a protective effect on CCS patients and SCFA producers have decreased in some CCS patients. Additionally, they improve health by boosting the immunological response of the host, preserving the integrity of the intestinal barrier by controlling the expression of tight junction proteins, and reducing blood lipid levels by preventing the production of cholesterol (David et al., 2014; Krishnan et al., 2015; Chambers et al., 2018). SCFAs play a protective role in atherosclerosis, while LPS stimulates body inflammation and accelerates the formation of atherosclerosis (Vinolo et al., 2011).

When butyrate-producing bacteria disappear, the gut barrier may become damaged, making it easier for microbial toxins like LPS to leak and cause inflammation by binding to Toll-like receptors. Patients with CAD have been found to have a higher LPS biosynthesis in the microbiome, which is connected to insulin resistance and abdominal obesity (Trøseid et al., 2013; Brandsma et al., 2019).

The synthesis of TMAO, a strong risk factor for the development of CAD, involves dietary choline, betaine, phosphatidylcholine, L-carnitine, and lecithin which are obtained from a variety of sources, including egg, fish, red meat, soybean, peanuts (Tang et al., 2013; Zhu et al., 2016). By producing choline and the intermediary molecule trimethylamine (TMA), the gut bacteria also contribute to the synthesis of TMAO. The capacity of the gut microbiota to synthesize choline via the phospholipase D (PLD) enzyme has just recently been discovered. The flavin-containing monooxygenase (FMO) enzyme metabolizes the microbiome-derived TMA molecule into TMAO in hepatocytes (Phillips et al., 1995). Firmicutes, Proteobacteria, Actinobacteria, and Bacteroidetes are the TMA producers. The production of foam cells is stimulated by the impairment of cholesterol metabolism in macrophages caused by TMAO-dependent activation of macrophage scavenger receptors and CD36 expression. The higher the TMAO production, the higher the CAD risk (Wang et al., 2011). In the context of increased intestinal permeability, TMAO is also linked to C-reactive protein (CRP), endothelial dysfunction, and elevated blood levels of the LPS endotoxin. It can also cause platelet hyperreactivity, which has an impact on the CAD progression (Al-Obaide et al., 2017).

The LDL-C level was lowest in CCS patients. The reason for the differences in LDL-C levels was that the majority of patients (96.67%) were treated with statins. The treatment with statins is associated with a lower prevalence of gut microbiota dysbiosis (Vieira-Silva et al., 2020). In mice study, statins could moderate gut microbiota by increasing the abundance of *Bacteroides, Butyricimonas,* and *Mucispirillum* (Kim et al., 2019). Moreover, the clinical response to statin therapy in individuals with CAD is associated with the gut microbiota. For instance, poor statin response is associated with a significant reduction in the number of beneficial bacteria (*Akkermansia muciniphila* and *Lactobacillus*) and an increase in the number of bacteria (Holdemanella and Facecallibacterium) (Wang et al., 2021). Statins have also been linked to anti-inflammatory and immunomodulatory properties (Lee et al., 2016).

The genus *Megasphaera* was strongly positively correlated with TG level and negatively correlated with HDL-C. López-Montoya et al. (2022) investigation confirmed our results, showing *Megasphaera* and *Escherichia-Shigella* were highly associated with atherogenic dyslipidemia patients, defined as both hypertriglyceridemia and low HDL-C. Moreover, one study has shown that patients with symptomatic stroke had an altered gut microbiota and defined *Megasphaera* as opportunistic pathogens (Zeng et al., 2019). In obesity and overweight patients, *Megasphaera* significantly increased relative abundance and correlated with low physical activity (Palmas et al., 2021). *Megasphaera* is involved in a mechanism that generates ammonia, which has negative consequences (Anand et al., 2016).

Hs-CRP can help in identifying chronic inflammation. Periodontal disease was linked to CAD and low-grade inflammation, increased CRP, and fibrinogen, according to De Oliveira et al.’s research (De Oliveira et al., 2010). There is a correlation between hs-CRP and CAD severity in CCS patients (Habib and Masri, 2013). Patients with myocardial infarction with elevated hs-CRP level (≥ 2 mg/L) were at higher risk of major adverse cardiovascular events and death (Carrero et al., 2019). In CCS patients of our study, the Lachnospiraceae, Peptostreptococcaceae families, and *Pediococcus* genus were positively correlated with hs-CRP, a finding that has not been reported before.

This study had some limitations, one of which was that the functional roles of the gut microbiota were not examined using a shotgun sequencing approach yielded more information due to its broader gene list and capacity to profile metabolic pathways. Moreover, the cross-sectional nature of this study makes it difficult to determine a causal relationship between changes in the gut microbiome and the disease. Other possible confounding variables that might have influenced the study’s findings were obesity, impaired fasting glucose, and polypharmacy.

## Conclusion

In conclusion, this study demonstrated a significant difference in the composition of gut microbiota between CCS patients, dyslipidemia patients, and healthy volunteers. The alpha diversity was lower in CCS and dyslipidemia patients than in healthy volunteers. The relative abundance of Proteobacteria, Fusobacteria, *Prevotella,* and *Streptococcus* was significantly increased while *Roseburia*, *Ruminococcus,* and *Faecalibacterium* were lower in CCS patients. In dyslipidemia patients, *Megasphaera* was strongly positively correlated with TG level and negatively correlated with HDL-C. Modification of gut microbiota is associated with changes in clinical parameters involved in the development of CAD in CCS patients.

## Data availability statement

The original contributions presented in the study are publicly available. This data can be found at: https://www.ncbi.nlm.nih.gov/bioproject/; PRJNA1000984.

## Ethics statement

The studies involving humans were approved by Ethics Committee of Chulabhorn Hospital and Srinakharinwirot University research ethics committees (IEC No: 174/2564, issued 22 July 2022 and SWUEC/E/M-100/2565E, issued 22 February 2023, respectively). The studies were conducted in accordance with the local legislation and institutional requirements. The participants provided their written informed consent to participate in this study.

## Author contributions

WL: Conceptualization, Data curation, Formal analysis, Investigation, Methodology, Project administration, Resources, Software, Validation, Visualization, Writing – original draft, Writing – review & editing. PP: Conceptualization, Data curation, Formal analysis, Methodology, Project administration, Software, Supervision, Validation, Visualization, Writing – review & editing. VM: Conceptualization, Validation, Visualization, Writing – review & editing. DS: Conceptualization, Data curation, Methodology, Project administration, Visualization, Writing – review & editing. EE: Conceptualization, Data curation, Supervision, Validation, Visualization, Writing – review & editing. MT: Conceptualization, Data curation, Funding acquisition, Investigation, Methodology, Project administration, Resources, Supervision, Validation, Visualization, Writing – review & editing.
